# Collectanea

**Published:** 1831-06

**Authors:** 


### COLLECTANEA.

Floriferis ut apes in saltibus omnia libant,

Omnia nos, itidem, depascimur aurea dicta.

### PATHOLOGY.

*Cases of Congenital Incontinence of Urine.* By John C. Otto, m.d., one of the Physicians to the Pennsylvania Hospital.

Case I. A lad ten years of age, laboured under incontinence of urine, and, as it had continued from his birth, his parents presumed it was a natural defect, that was beyond the reach of medicine. Possessing very delicate feelings, it was supposed his sense of shame might be addressed to some purpose, but it was in vain; and very moderate correction was resorted to without any advantage. Nothing further had been attempted by his parents, except restricting him somewhat in his drink, especially in the evening. He had arrived at a period of life when his deplorable state was obvious to him, and lamented bitterly his situation. He was strong, of a florid complexion, and had ever been remarkably healthy in other respects; his desire to make water had always been very frequent and urgent, and he discharged but a small quantity at a time. After he was four or five years old, he never wet himself in the day time, if he could retire immediately to a suitable place to void his urine, as soon as the desire occurred, for the urgency was always very great, and his powers of retention small and of short continuance. He passed very rarely a night without wetting his bed, and never two in succession, although great attention was paid to his making water when he went to bed, again when the family retired, most commonly once during the night, and always very early in the morning. I told his mother the case ought not to be considered incurable, and, should every attempt to give relief fail, he would not be in a worse situation than at present; that he might be essentially benefited, but, should there be a want of success, she would have the consolation of having used such remedies as were supposed best calculated to cure him. I directed an ounce of the leaves of the uva ursi to be simmered in a pint of boiling water five minutes, of which he was to take a wineglassful four times a day; and, in order that the virtues of the medicine should be principally extracted, it was to be prepared twenty-four hours before giving it, and decanted as used. He was likewise to take fifteen drops of the muriated tincture of iron three times a day, in a sufficient quantity of water, and to have a gallon of cold water dashed on the perineum and nates morning and evening. He improved rapidly under the treatment, having wet his bed for the last time on the 18th of October, not having done it for the previous eleven nights. The cold water, on which I had placed some reliance as a tonic, was not used at all; it was omitted at first from causes that were not satisfactory to me, and, as he had become so much better when I was informed of it, the employment of it was not pressed. The urgency of making water gradually lessened, and the power of retention improved, so as to become natural in both respects. Although he was entirely restored in so short a time, the treatment was continued three months altogether, as the incontinence was congenital, and he has remained ever since free from his distressing malady.

Case II. October 5th, 1829. J. M'G., aged twelve years, resides in one of our public charitable institutions, where he has been almost five years, during which time he has enjoyed good health, but subject to an incontinence of urine. He wets his bed about once a week; formerly he did it much more frequently; has a frequent desire to make water, and discharges but little at a time; and, unless he can retire to a convenient place soon after the feeling occurs, he occasionally wets his clothes in the day time. He was ordered to take a wineglassful of the decoction of the uva ursi, prepared as in the former case, three times a day, and ten drops of the muriated tincture of iron as often. His complaint fluctuated, but on the whole he improved, especially in being able to retain his water at night, but the change for the better was slight in the day; from which circumstance there was some reason to suppose there was a want of attention to cleanliness on his part, especially as he appears not to have much delicacy of feeling. The treatment was continued, and in addition a large blister was applied to the sacrum, which drew well; but three others, which were applied afterwards in succession, as soon as the parts admitted of it, occasioned but a slight discharge, and not much irritation; he, however, improved somewhat under this plan. On the 15th of December, I substituted the tartar emetic-ointment for that of the cantharides, and it produced the usual effect, but no further amendment followed its use, after a sufficient perseverance in it. On the 10th of January of the present year, the whole treatment was abandoned, and I directed a decoction of the leaves of the smooth leaved or upland sumach, the rhus glabrum, an ounce to the pint of water; of this a tablespoonful was to be given three times a day. This plan was pursued with some slight amendment, until the 14th of March, when he was directed to take, in addition to the decoction, fifteen drops of the oleum terebinthinæ, which were soon increased to twenty, three times a day; and, on the 18th, a scruple of the pulverized leaves was substituted for the decoction. This treatment was continued, and he was so much better than he had ever been, if not entirely restored, that he left the institution on the 20th of May to be bound as an apprentice.

Case III. September 30th, 1829. M. N., aged nine years, frame stout, habit full, and intellect very dull. She resides in the same institution with the preceding case, where she has been for the last three years, during which time she has been healthy and strong. Ever since her admission she has been subject to incontinence of urine, which probably has continued from her birth, as she has no recollection that she was at any time exempt from it, nor has any thing ever been attempted to relieve her, except restricting her in the quantity of her drink, which was the case with all the others in her situation, but it was to no purpose. She rarely misses wetting her bed, although every attention is paid when she goes for the night, and she is taken up when the elder part of the family retires. She makes water frequently and in a small quantity, and is not able to retain it long, when the desire to discharge it takes place. She very rarely wets herself in the day time, unless spoken to sharply, or is suddenly agitated; then she invariably passes some water, whoever may be present, being entirely deprived of the power of retaining it. She was directed to take a wineglassful of the decoction of the uva ursi, prepared as previously mentioned, and five drops of the muriated tincture of iron as often. As no improvement took place in four days, the dose was increased to ten drops, after which she mended rapidly, and did not wet her bed for several months, after the 20th of October, but the remedies, from the violence of the case, were continued three months altogether. Since then she has a few times, but very rarely, wet her bed, and it is probable that the occurrence may be ascribed in part to her great dulness, and want of delicacy of feeling.

Case IV. September 30th, 1829. W. U., aged ten years, has been in the same institution two years and four months, and has had an incontinence of urine ever since his admission; he very rarely misses wetting his bed, and appeared rather worse than usual this summer. Every attention is paid when he retires for the night, and he is taken up to make water when the family go to bed. He wets his clothes likewise in the day time so frequently, that even with great care he becomes offensive. The same quantity of the decoction of the uva ursi, prepared as in the other cases, was directed to be taken by him three times a day, and eight drops of the muriated tincture of iron as often. Finding but little improvement in his condition, I applied, on the 3d of November, a large blister to the sacrum, and three others were applied in succession, but only the first drew powerfully, and was followed by a copious discharge. There seemed to be some amendment in his condition, but it was not considerable. The quantity of water he has always made was very great, approaching to what would be voided in a mild case of diabetes, with a corresponding degree of thirst. For a month longer the same medical treatment was continued, except that for the blisters the tartar emetic-ointment was substituted, which kept up a considerable irritation over the sacrum and neighbouring parts by a succession of pustules. Perceiving that no benefit was derived from this plan, the whole of it was laid aside on the 10th of January of the present year, and a decoction of the leaves of the rhus glabrum, an ounce to the pint of water, was ordered, of which a tablespoonful was to be given three times a day. By the 14th of March a slight improvement had taken place; but I thought further efforts should be made, and prescribed ten drops of the oleum terebenthinæ, three times a day, without omitting the other medicine. In the course of a week he had become much better than I had ever seen him, and the dose of the turpentine was increased to fifteen drops. On the 28th of the month fifteen grains of the powdered leaves were substituted for the decoction, which treatment has been continued ever since. His situation has very materially been improved, but he is not entirely well. His attacks seem to come on in paroxysms, being unwell for two or three nights in succession, with a considerable interval of entire relief.

This disease exists, it is probable, more frequently than is commonly imagined, as all those whom I have visited, when it was congenital, were presumed to be beyond the power of medicine, and were left for years to the operations of nature: my attention was directed to them rather accidentally. There being merely an inability to retain the urine long, and no constant irritation or excessive discharge, there is no peculiarity in the appearance of those who have this affection, nor are their physical powers in the least impaired. It seems to depend on an irritable state of the bladder, which occasions it to contract when its contents are small, and an unusual relaxation of the urethra or sphincter vesicæ, shewing its inability to resist the expulsive efforts of the bladder. In the third case, whatever excited agitation or fear, which, it is known, are calculated to relax the sphincters, produced a discharge of urine, which shews how much debility has to do with this affection. It occurs sometimes in those who are not accustomed to it, when the system becomes prostrate by disease. In none of them was there a sense of fulness or distention in the region of the bladder, nor could any examination externally discover any tumefaction of it.

My object was to give tone to the urinary organs, and with this view I directed the muriated tincture of iron. To this was added the uva ursi, which seems to be valuable as an astringent, and has some peculiar operation on the diseases of the urinary system, having been used from remote antiquity as a diuretic, and is employed by the moderns with singular advantage in calculous affections, nephritis, diabetes, catarrus vesicæ, and other affections of these parts. I had besides completely cured, many years ago, a case of incontinence of urine, of several months standing, that was the effect of gonorrhœa, by giving twenty grains of the powder three times a day, until an ounce was taken.—*North American Med. and Surg. Jour.*

*Gonorrhœa produced by the Lochia.* In the fifth volume, page 203, we noticed a case of gonorrhœa produced in a man by cohabiting with his wife too soon after delivery, and also an instance in which an ulcer of the prepuce was induced by the same cause. We took that occasion to express our views respecting the nature of gonorrhæa, and are happy to be able to adduce in support of them the following fact, communicated to us by our friend, Dr. J. K. Mitchell, of this city.

A gentleman, of unquestionable moral character, and who has always lived happily with his wife, was affected by all the usual symptoms of gonorrhœa, because of a too-early intercourse after delivery. The purulent discharge from the urethra was followed by a glandular swelling in the groin, which terminated in an abscess.—*American Journ. of Med. Sciences.*

---

*Case of Colica Constipata removed by Inflation.* By John King, Jun., Surgeon, Irvine.

The importance of inflation as a remedy for obstruction of the bowels, appears to me not to be sufficiently appreciated at the present day. It was first recommended by Hippocrates for the removal of intestinal obstruction; in more modern times, it has been resorted to by Hoffman and Haller; and notwithstanding the neglect it has since experienced, I cannot but regard it as worthy of an eminent position in the list of therapeutic agents. The treatment usually prescribed in cases of ileus or colica (without inflammation) is very discordant; as witness warm baths, fomentations, injections of warm water and oil, rubefacients, and blisters: contra, cold effusion and immersion, freezing lotions, pounded ice and snow; not to mention emetics, purgatives, and mechanical distention by warm fluids, quicksilver, gold and silver balls, &c., and when all these remedies have failed, bloodletting, tobacco in infusion and smoke, and lastly, gastrotomy. Yet this simple means of inflation, although probably the most powerful, and the least dangerous, is entirely overlooked. It paralyses, as it were, the constricted fibres of the bowels, and may be used in the following cases, if not with complete success, at least with advantage, viz. the various kinds of colic, proceeding from torpidity, spasmodic constriction, viscid meconium in new-born infants, impaction, bezoards, and other intestinal concretions, volvulus or intus-susceptio, and some cases of hernia. It was a happy thought of those who hit upon this means in the hour of danger, after all their other efforts had proved nugatory. For although tobacco, which is often used as a last resort, sometimes is successful, it is not uniformly so, and it too often happens, that the patient, rather than undergo a repetition of it, beseeches to be allowed "to die in peace." We may also observe the hesitation with which the practitioner has recourse to it; not only because of its doubtful efficacy, but on account of the danger there is of greater exhaustion being produced by it. I take the liberty of giving one case, as I conceive it may give some idea of the power of inflation.

In September 1829, I was requested to visit Mrs. G. æt. twenty-six, of rather delicate frame. On the night previous to my visit, she experienced an uneasy sensation in the region of the stomach; for which, she took eight grains of calomel combined with a half-drachm of compound powder of jalap, without any impression on the bowels. During the night this uneasiness increased to an almost intolerable pain, accompanied with obstinate vomiting, which continued till the evening, when I saw her. In the course of the day she took two doses of castor oil, and received five injections. When I entered the apartment, she was sitting near the fire, and her body bent forward; the face was wan, hollow, dejected, and of a dingy yellow colour; the surface of body and extremities inclining to cold. Pulse eighty, soft and much compressed; tongue, at the back part, covered with a brownish coloured mucus: she had obtained no alvine solution for six days. She took no notice of my being present, or of any thing going on around her, but informed me, when questioned as to the seat and kind of pain, that it was of "a violent screwing nature, working between the stomach and navel," coming on in paroxysms, and ending in, or producing vomiting. I ordered the warm bath, and gave a teaspoonful of laudanum with compound spirit of lavender, which was soon afterwards vomited. Upon this, an effervescing mixture was given, then five drops of croton oil with some laudanum; and in about three quarters of an hour, five drops more without laudanum: but each in its turn was rejected, with a quantity of yellow-coloured fluid. It was at this time I first thought of inflation. For this purpose, I procured a pair of common bellows, and securing the bladder of a clyster bag to the nozzle of the bellows, the pipe was introduced into the rectum, while the patient lay on her right side, and the bellows was commenced being wrought. As soon as the air entered the rectum, the effect was immediate and satisfactory; the countenance lost its anxiety, the eye brightened, and the patient said she felt quite relieved. A gurgling noise was heard in the bowel, with an escape of fœtid air; and in about a minute from the time the air began to enter the rectum, she requested to be allowed to go to stool. She had a copious dejection, and a good night's rest; and next morning complained only of being much enfeebled, but was otherwise well.

I was deeply impressed, about five years ago, with the fatal result of a case of intus-susceptio, in a fine robust infant, six months old; which was supposed to proceed from the effects of half a teaspoonful of some syrup of poppy, made, as is commonly done, with opium, given for the purpose of procuring sleep during the period of teething. About eight hours after it was given, the child began to cry vehemently, having appeared restless and uneasy for several hours previously. Early in the forenoon, it passed a very scanty stool, streaked with blood; soon after this, vomiting commenced, which continued until the little sufferer sunk. Is it unreasonable to imagine that, if inflation had been used in this case, the result would have been otherwise? I was hereby shewn the necessity of seeking more powerful means than fluid injections, et cætera: and I hope, as I firmly believe, that inflation with common air is the necessary desideratum. I conclude with Dr. Cheyne, that "a man dying of ileus, presents one of the most pitiable sights in nature; and a leading object of this paper is to remove a part of the horrors of the scene, by withholding many of the bitter doses which are forced upon him by the solicitude of his friends, and officiousness of his physician."—*Glasgow Med. Journal.*

---

### PRACTICAL MEDICINE.

*Tetanus cured by Injection of Opium into the Veins.* MM. Percy and Laurent have cured three Russian soldiers affected with tetanus by injections of opium into the veins.—*Journal des Progrés.*

---

*Tetanus cured by Injections of Datura Stramonium.* Twenty-four grains of datura stramonium, dissolved in half an ounce of water, or a strong decoction of this plant, have been successfully injected into the veins in some cases of tetanus. According to MM. Percy and Laurent, the authors of these trials, the happy effects of this medication should be referred to the production of a kind of general paralysis favorable to the cure of this disease.—*Ibid.*

---

*Employment of Calamine to prevent the Pits of Confluent Smallpox.* Mr. George, of Kensington, has recently published in the Medical Gazette, some interesting cases, which shew the possibility of preventing, to a certain extent, the frightful deformity which not unfrequently arises from confluent smallpox.

One patient was twenty-four years of age. About the tenth day he was much exhausted by the disease. The cuticle, from adhering to the bedclothes, was abraded to the extent of six or seven inches on each hip, and to the same extent in each ham and on the back. Mr. George covered the exposed surfaces, and kept them constantly covered, with the prepared calamine. In four days the cuticle was every where restored, and the patient recovered more rapidly than usual. There was afterwards not a single pit to be observed where the cuticle had been so extensively removed, and even the immediate surrounding pustules, which were unavoidably covered with the powder, had not destroyed the cutis.

Mr. G. had previously conceived that any successful mode of local treatment would disarm the malady of half its loathsomeness, and consequently control the constitutional disturbance. The immense surface of exposed cutis, the discharge, the exquisite sensibility, all reminded him of the appearance and effects of a scald, and, reasoning by analogy, he tried the same mode of treatment, with the beneficial effect above stated.

SURGERY.

*Extraction of Foreign Bodies from the Eye.* In Livonia, and in some parts of the province of Frankfort, especially the circle of Sternberg, the country people have a peculiar method of removing foreign bodies from the eye, in which they shew much dexterity: they lick out the eye with their tongue, while they catch hold of the eyelashes of the lid under which the foreign body lies, and thus remove it.—*Jüngken's Lehre von den Augenoperationen*, p. 493.

---

*Proposed Operation for Lagophthalmia.* After describing the common operation, and the uniform want of success that attends it, Dr. Jüngken says, "I have resolved, in the next case that occurs, to perform the operation in the following manner: I shall cut round the scar with the scalpel, in the manner already mentioned, and extirpate it, enlarging the wound enough to give the eyelid sufficient length, and bring it into its natural position. For the sake of greater security, the size of the wound may be drawn upon paper, and cut out. In case the disease is in the under eyelid, I shall cut round a piece of skin on the side of the cheek; but if in the upper eyelid, I shall then take it from the forehead: this piece of skin is to be of the exact shape of the wound, and to be connected with the surrounding skin by a narrow bridge. I shall then dissect out this piece, with as much cellular substance as possible. The bridge, or slip of skin, must be so long, that the principal piece may be turned over to the wound, and laid upon it, without destroying its original connexions. I shall then stop the hemorrhage in the most careful manner with cold water, remove all the coagulated blood, and then, by turning round the slip of skin, place the vicarious piece in the wound, in such a manner that the edges and surfaces of the wound and its new covering may exactly fit, the external skin still remaining outside. The union must take place by the application of the sutura nodosa, in order that the piece of skin may not be able to shrink; the rest of the bandage should consist of a pledget and sticking plaster.

"The bridge is to be retained until we have reason to suppose that an organic union has taken place between the piece of skin and the subjacent part: it is then to be divided with a scalpel upon a hollow sound, close to the edge of the piece of skin, and the remainder of the bridge is to be returned to the spot from which it was taken, and fastened with sticking plaster, so that union may again take place there. The threads which formed the ligature are to be taken out at the proper time, and the bandage is then to be continued by sticking plaster alone, until entire cicatrization takes place. The wound caused by the abstraction of the piece of skin is to be closed as tightly as possible by sticking plaster, that it may heal with a small scar.

"It might, perhaps, be possible in this manner to effect a radical cure of lagophthalmia; and the scar which would remain on the cheek or forehead, would be far from deforming the patient so much as a shortened eyelid.

"In conclusion, I request my professional brethren to look upon this proposed method of treatment in the light of a proposition as yet untried, and to subject it to more accurate examination, especially by actual experiment."—*Ibid.* p. 267-8.

Dr. Jüngken, in his preface, gives the following account of his own experiments on this subject:

"In the operation for shortened eyelids, I have proposed, at page 267, to effect a radical cure by lengthening the shortened eyelid, through uniting to it, by the process of healing, a piece of skin from the cheek or temple. Since writing this passage, I have twice operated in this manner upon an ectropium caused by shortening of the external skin of the eyelid: the first time on a scrofulous boy, the outer skin of whose right lower eyelid had been so destroyed by gangrenous erysipelas, that complete eversion had taken place; the second time on a mechanic, who, in consequence of a scorpion's bite at Naples, had suffered from gangrene of the left lower eyelid and the cheek; after which, the external part of the eyelid became so shortened, that in his case likewise complete eversion existed, and the inflamed and swollen conjunctiva of the eyelid was seen turned outwards. In other respects, the patient, when he came under my care, was strong and healthy. In both cases, the operation failed entirely.

"I was much pleased, however, to hear, a short time since, from Dr. Fricke, of Hamburg, during the stay that he made in Berlin, that he had performed this operation with success: he has described it in an interesting essay, entitled "The Formation of New Eyelids, or Blepharoplastics; by Dr. Fricke. Hamburg, 1829."

---

*Lithotrity.* Dr. Civiale recently laid before the French Academy a report of various cases of lithotrity. The process sometimes has failed, but the failure has always, in the opinion of Dr. C., depended upon the imperfection of the instrument employed. Lithotrity has been successfully practised in 163 cases, according to the plan which he adopts and recommends: 152 of these cases were operated upon by himself.

---

*Acupuncturation.* The last number of the Edinburgh Medical Journal contains an interesting paper on this subject, by Dr. Tatam Banks. His experience accords with that of Dr. Elliotson and Mr. Churchill, that acupuncturation is chiefly useful in chronic cases. Five cases are related in which this treatment was adopted: in the first, the patient had for many years been subject to severe rheumatic pains of the right hip, thigh, and leg. Various modes of practice had been adopted without avail. Dr. B. inserted "five needles into the anterior part of the glutæus medius and maximus, and four into the tensor vaginæ femoris." In two minutes, the patient declared the hip was free from pain, and eagerly requested the back part of the thigh and leg to be punctured. Dr. Banks accordingly introduced five needles, about an inch deep, into the biceps flexor cruris and vastus externus, and three into the gastrocnemius externus, about an inch and a half apart. The muscles were affected with powerful spasm, but the pain entirely vanished. The man, who had before been lying in bed, and quite unable to turn himself, "felt certain he could now walk, and in an instant was out of bed, and began striding and jumping across the apartment." The needles were left in about fifteen minutes: but little pain was felt when they were introduced. The patient suffered no relapse. The second, third, and fourth cases were of a similar nature, and the same practice was equally successful as in the former. We give the fifth case without abridgment:

A stout healthy-looking man had been, for nine days, labouring under Neuralgia Faciei, generally named Tic-douloureux. The symptoms indicated the disease to be seated in the portio dura of the seventh pair of nerves of the right side, the divarications of pain corresponding exactly with its course and communications. The muscles of the face were affected with convulsive twitchings, and he appeared to suffer the most pungent agony. But he complained most bitterly of deep-seated pain at the under and back part of the ear, where the portio dura passes out of the bone through the foramen stylo-mastoideum. The pain came on in paroxysms. During the first three or four days, a paroxysm usually occurred about six in the evening, and continued violent between two and three hours; but latterly they have recurred so frequently, during both day and night, as scarcely to leave him the enjoyment of half an hour's ease. He attributes his complaint to getting his feet wet, and remaining too long in his wet shoes and stockings. On my first seeing him, he strenuously refused to have the needles inserted, and wished me to prescribe some medicine. In the present instance the brain, I fancied, did not escape disease; for weight and pain were felt in the head, with slight giddiness; the pulse was full and accelerated, and the bowels regular. Conceiving the neuralgia facialis might probably proceed from determination of blood to the brain, I directed bleeding, both general and local, and aperients, together with a blister to be applied on the back of the neck. By these means the head was entirely relieved; but the anguish produced by the tic-douloureux continued as great as ever. I now prescribed one drachm and a half of the subcarbonate of iron four times a day, and persisted in its use two days, without the least abatement of the symptoms. I next ordered the extract of stramonium, but without advantage; and did not succeed in allaying the pain until I had recourse to belladonna, from which medicine he certainly derived, for some days, great benefit. But a paroxysm of most acute pain suddenly occurring when I happened to be present, quite disheartened him; and he was now, upon my relating one or two cases in which I had seen acupuncture serviceable, readily induced to try its effects on his own person. I accordingly lost no time in introducing five needles in the several directions where the pain darted, in the right side of the face and temple. Before I had well finished doing so, he joyfully exclaimed, that the pain of his face had completely gone. On the following morning, when I called, he told me he had suffered no pain the whole night; and appeared so satisfied that he owed his present ease to the good effects of the operation, that he earnestly begged of me to repeat it. It may appear strange to say, that I did so merely to gratify him; but such was the fact. Although three months have transpired since this case was under my care, yet no tendency whatever to this excruciating disease has again manifested itself.

In no instance have I known any untoward symptom occasioned by acupuncturation. I cannot affirm that no pain is produced by the operation; but certainly it is very slight. Where a syphilitic taint is connected with rheumatism, of course acupuncturation alone will prove unavailing.

---

*A Case of severe Scald treated by the Nitrate of Silver.* A little child, five years old, was pushed backwards by another child, whilst naked, into a large pan of scalding water which had been just taken off the fire. It was taken out as quick as possible, and yeast was applied upon the injured parts. It was visited one hour after the accident. The whole of the back, as high as the shoulder-blades, and as low as the middle of the thighs, was found severely scalded, the cuticle removed from some parts, and in other parts raised into large vesications. The whole of the belly, the penis, scrotum, and thighs, were also in a similar state, but not so severely scalded as the back. An opiate was administered, and the yeast was removed with a sponge and warm water; it was well that no oily application had been used, as its removal would have required more trouble and have given more pain. The loose cuticle was removed with that of all the larger vesications, and the small ones were punctured, so that a clear surface was presented to which the nitrate of silver might be applied.

The whole surface was then moistened with pure water, and a long stick of the nitrate of silver was applied flat, once over the whole surface, and a little on the surrounding healthy skin. A little linen, just moistened, was then past over every part to diffuse the nitrate of silver, so that no spot might be left untouched. The child cried much less than was expected when the nitrate of silver was applied on the denuded cutis. The back, on which the child would have to lie, was then covered with neutral ointment spread upon linen, secured by a bandage. The thighs and belly were left exposed to the air to form an adherent eschar, being defended by a fracture cradle.

On visiting the child about eight hours afterwards, it was reported to have fallen asleep in a quarter of an hour after the application of the nitrate of silver, and to have complained of no pain since. There appeared no constitutional disturbance.

The very first morning after the accident, this little patient was turned on his side, enjoying some playthings with several play-fellows who were by the side of his bed. One part on the side of the thigh was much swollen and inflamed. It was discovered that the nitrate of silver had not been applied upon it. The whole of the belly and the other parts of the thighs exposed to the air looked very well, with scarcely any vesications; the eschars were removed in two places where the tapes of the bandage had crossed the belly: these parts were now defended by means of a small plaster of neutral ointment spread on linen. On some parts the eschars were floating on the serum: these afterwards became adherent. The scrotum and penis were much swollen, but gave no pain. The nitrate of silver was applied on the part not attended to at the first dressing.

On the second day the child was going on well; some of the eschars were becoming adherent; the scrotum and penis continued much swollen, but there was scarcely any pain, and that on the belly. There was slight heat of skin, and the tongue was a little loaded. A purgative with senna and salts was given.

On the third day nearly the whole of the eschars were found to be adherent, and the scrotum and penis less swelled.

On the fourth day the eschars were quite adherent on the belly, and the penis and scrotum were of their natural size.

On the fifth day the plasters of neutral ointment were removed from the back, which presented an appearance of a recently blistered surface, in a healing state, with some loose cuticle partially attached; there was no appearance of suppuration.

In several days more the back was healed, except in two or three small parts, which were scalded more deeply than the rest, and were covered over with coagulable lymph, not the least suppuration having taken place. The eschars were peeling off the belly, leaving the subjacent surface quite healed.

On the tenth day this little patient was out of doors, and on the twelfth at school, every part being quite healed.

Mr. Higginbottom, in his Essay on the use of the Nitrate of Silver makes the following observations on burns and scalds:

"I have found that, by slightly passing the nitrate of silver once over a burnt surface, the pain is increased for a short time, but then totally subsides, vesication appearing to be prevented; the black cuticle peals off in a few days, leaving the part well. In cases in which the cuticle has been removed, the nitrate of silver applied on the surface induces an adherent eschar, and prevents the consequent ulceration."—P. 149.

"I have not had an opportunity of using the nitrate of silver in very extensive recent burns, but I can have no doubt of the benefit that would accrue from it. It should, I think, be applied over the whole surface of the burn or scald, once only, as in external inflammation; then the parts most severely burnt should be covered with lint, and the whole of the burnt surface with the neutral ointment spread on linen, a bandage being applied to retain the dressings in their places. I should expect that the inflammation would be checked, and the consequent vesication, ulceration, and sloughing, in a great measure prevented, except in those places where the fire had actually destroyed the parts deeply. I should not examine the parts again before the fourth or fifth day; and if the dressing adhered, I would let them remain during another similar period. The application of the nitrate of silver should be repeated in the same manner, as might appear to be required. I think the burn would then be limited in its extent, and would consequently be less dangerous; for the danger is generally in proportion to the extent of surface destroyed. The nitrate of silver has certainly the property of removing the irritability of the whole surface to which it is applied, and cannot add much to the pain of the burn itself."—P. 150.

These anticipations appear to be correct when the nitrate of silver is applied to a burnt or scalded surface from which the cuticle is not removed. It has the immediate effect of subduing the heat or burning pain, preventing vesication, and causing it to terminate by resolution.

When the skin is denuded of the cuticle, and the nitrate of silver is applied, this most irritable and inflamed surface is converted into an insensible covering, which remains adherent until the inflammation is gone, and the new cuticle is formed underneath, at which period it loosens and drops off.

The application of the nitrate of silver is equally efficacious whether the burn or scalded surface be afterwards exposed to the air or covered by the neutral ointment. In the first case an adherent eschar is formed in two or three days; and in the second the effects of the nitrate of silver appear to continue for four or five days, producing a constant flow of serum, which continues until all the inflammation, irritation, and pain, are gone. It is possibly of little consequence which plan is adopted, as both are healed about the same period. The adherent eschar would be preferable in parts exposed, as the face and neck; or the chest, belly, or legs too, if defended by a fracture cradle, and the patient in bed.

The advantages of the nitrate of silver in the treatment of burns and scalds appear to be of the very first importance. We have at once a covering for the injured and very irritable surface, superior to any other formed and composed partly of the very surface itself. The nitrate of silver acts as an anti-inflammatory agency, both immediately and for several days after its application.

It may be safely applied over the head, chest, or abdomen, and it is not, like arsenic, and some other remedies used externally, liable to be absorbed into the system.—*Edinburgh Med. Journal.*

*Fracture of the Patella.* In this case reunion is opposed by the four following circumstances: 1st, The difficulty of retaining the broken surfaces in contact; 2d, The absence of vascular parts, or indeed any parts at all, on the inner side of the bone, to throw out an effusion of organizable matter for connecting the fragments in the first instance, before they are joined together by their own process of ossification; 3d, The presence of the fluid of the joint, which is secreted in greatly increased quantity by the irritation of the injury; 4th, The spongy texture of the bone concerned, which is observed to have less power of ossific effusion than the dense portions of the same tissue.

Four cases of this accident have presented themselves to our observation. The first of these was that of a man, æt. thirty-five, from Rothsay, who applied at the hospital on the 13th of June, immediately after having fractured the patella transversely by falling from a cart. The proper bandages were applied; but he refused to remain in the house, being anxious to return home without delay. The second was of a year or more standing, and the patient, a young woman, came from the neighbourhood of Melrose, anxious to have some operation performed for her relief. On examination, it appeared that the fragments remained perfectly moveable, so that, when the limb was extended, they could be placed completely in contact, and, upon its being bent, separated to the extent of three or four inches. The limb was consequently very much weakened, and she was therefore desirous of relief. I recommended her to procure a bandage, which should have the effect of retaining the two portions of the patella completely, or nearly, in contact, and this was effected by means of two broad circular collars of leather, which being laced tight round the thigh, were then drawn together by straps at the sides of the knee and patella. She went away very well pleased with the improvement in her condition, and I have heard nothing of her since. The third was also an old case, the patient being Duncan Campbell, æt. fifty-three, from Leith, who was admitted into the hospital on the 30th of December, for fracture of both bones of the leg. The knee presented a very unusual appearance, and, upon examination, it was found that there existed not only an old fracture of the patella, but also a subluxation of the joint outwards. These injuries were stated to have been sustained nearly forty years ago in leaping over a high wooden fence. The fragments of the patella were several inches distant from each other, and greatly increased in size; indeed, the upper one was nearly three times as large as it ought to have been. Notwithstanding all this, he had been able to discharge the duties of an infantry soldier for thirty years.

The fourth case was that of George Sinclair, æt. thirty-seven, who was admitted on the 1st of January, on account of a transverse fracture of his left patella, which he had sustained the same evening by falling down a stair. The fragments were considerably distant, and a large effusion had taken place into the cavity of the joint.

The limb was kept straight, and a pillow placed under the foot, to relax still farther the rectus muscle. Discutient lotions were applied to the knee, and circular bandages above and below the patella, with connecting bands on each side of it. The effused fluid was very gradually absorbed, and it was long before the fragments could be brought together. The patient is still in the house, but will soon, I trust, be discharged with little imperfection of the limb. (From Mr. Syme's *Hospital Report; ibid.*)

